# Understanding the Role of pH Regulation and Neutralizing Agents in Organic Acid Production and Growth of *Aspergillus oryzae*

**DOI:** 10.1002/bit.70091

**Published:** 2025-10-31

**Authors:** Lukas Hartmann, Mark Christopher Martin, Anke Neumann, Dirk Holtmann, Katrin Ochsenreither

**Affiliations:** ^1^ Karlsruhe Institute of Technology Karlsruhe Germany; ^2^ University of Applied Sciences Kaiserslautern Pirmasens Germany

**Keywords:** malic acid, neutralizing agent, organic acid, pH control, pH level, respiratory activity

## Abstract

With growing interest in the valorization of renewable resources, the microbial production of organic acids using *Aspergillus oryzae* has gained attention. However, process parameters such as pH and neutralizing agents remain insufficiently understood. We investigated the effect of pH and different neutralizers on the production of malic, succinic, fumaric, pyruvic and citric acid and fungal growth using offline sampling and online monitoring of respiratory activity. Neutralizers included NaOH, Na_2_CO_3_, KOH, Mg(OH)_2_, Ca(OH)_2_, and CaCO_3_ and were compared to the conventional use of excess CaCO_3_. Using Na_2_CO_3_, malic acid reached 33.18 g L^−1^ with a yield of 0.54 g g^−1^ from glucose and a productivity of 0.14 g L^−1^ h^−1^. KOH enabled the highest citric acid concentration of 9.12 g L^−1^ with 0.18 g g^−1^ and 0.04 g L^−1^ h^−1^. At controlled pH with NaOH, pH 7.00 resulted in 39.14 g L^−1^ malic acid with 0.60 g g^−1^ and 0.17 g L^−1^ h^−1^. Citric acid peaked at pH 5.50 with 20.18 g L^−1^, 0.36 g g^−1^ and 0.09 g L^−1^ h^−1^. Under dynamic pH conditions, acidification suppressed the production of most acids, while citric acid was produced exclusively at low pH. Off‐gas analysis at controlled pH revealed increased respiratory activity under acidic conditions, indicating active pH homeostasis. Furthermore, we detected a nutrient limitation via respiration monitoring in a medium widely used for decades, uncovering untapped optimization potential in previously published studies. These findings highlight the importance of pH and neutralizer selection for improving microbial organic acid production.

## Graphical abstract

We show that pH and neutralization strategy strongly influence the growth, pellet morphology, and organic acid production of *Aspergillus oryzae*. Acidic and alkaline conditions lead to hairy, dispersed pellets, while near‐neutral environments favour compact pellet formation. Both pH and neutralizer type steer the spectrum and relative abundance of produced organic acids, underscoring the role of buffering conditions in shaping fungal metabolism.

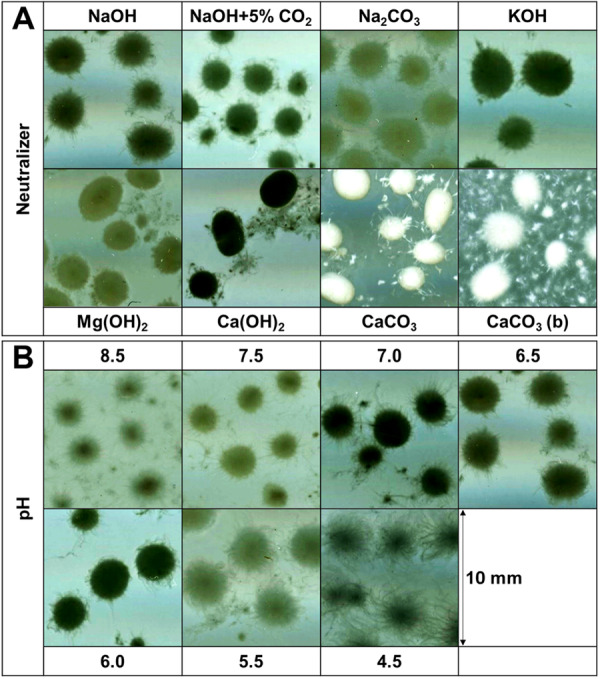

## 1 Introduction

Fungi, as natural decomposers, exploit a wide variety of organic substrates and are utilized for large‐scale bio‐based production of enantiomerically pure organic acids (Hu et al. 2014; Kuenz et al. 2012; Mohamad et al. 2010; Znad et al. 2004). *Aspergillus oryzae* (*A. oryzae*), as one of the most widely utilized *Ascomycetes*, has a fully sequenced genome and is employed in various GRAS recognized applications, including as a probiotic, a feed additive, for the production of fermented foods, as an expression platform for hydrolytic enzymes and for the production of organic acids (Francis et al. 2003; Higginbotham et al. 2004; Lee et al. 2005; Lynn et al. 2013; Machida et al. 2005; Payne et al. 2006; Yang et al. 2017). In 2004, the U.S. Department of Energy identified 12 key building blocks derived from biobased feedstocks as promising platform chemicals (Werpy et al. 2004). *A. oryzae* produces three of these platform chemicals: malic, fumaric and succinic acid. (Kövilein et al. 2022).

Until today, malic acid has been predominantly manufactured via petrochemical processes, with an estimated global production of 1.05 × 10^5^ t in 2023 (ChemAnalyst 2024). These routes involve the oxidation of fossil‐derived butene to maleic anhydride, followed by hydrolysis to maleic acid, isomerisation to fumaric acid, and subsequent hydration to racemic malic acid (Kövilein et al. 2019; Winstrom et al. 1968). On a smaller scale, l‐malic acid is enzymatically synthesized through the hydration of fumaric acid using fumarase (Ortiz et al. 2017). Given that the global market value of malic acid is estimated at $0.92 billion in 2023 and projected to grow to $1.41 billion by 2032, microbial production utilizing renewable resources is expected to play a meaningful role in addressing the rising demand amidst constraints on fossil resources (Rittmann 2008; Zion Market Research 2024).

This increasing demand underscores the importance of optimizing microbial production processes, including strategies to manage challenges in pH regulation during organic acid production. To compensate pH drops during organic acid production, in media presented buffers or dosed neutralizers are used. Buffers like poorly soluble CaCO_3_ and MgCO_3_ are easy to handle, precipitate dissociated organic acids as salts, simplify product separation and mitigate product inhibition (Kövilein et al. 2021). Furthermore, CO_2_ formed from the acid‐carbonate neutralization is suspected to accelerate carboxylation reactions such as the conversion of pyruvic to oxaloacetic acid, enhancing the rate‐limiting step in the reductive TCA (rTCA) branch (Brown et al. 2013; Knuf et al. 2013). However, anions in calcareous salts are resolubilised with H_2_SO_4_, precipitating environmentally concerning gypsum of low economic value. (Ding and Ye 2023; Yang et al. 2011). Poorly soluble buffers increase viscosity, impairing mass transfer in submerged cultures (Kim et al. 2012; Mooney 1951). In addition, the pH during cultivation mirrors the neutralization equilibrium of organic acids and carbonates, preventing tailored pH levels (Salek et al. 2015). In contrast, individual pH levels are adjusted using water‐soluble Na^+^ or K^+^‐containing carbonates and hydroxides as well as poorly soluble neutralizers like Ca(OH)_2_ and CaCO_3_ (Balakrishnan et al. 2020; Brink et al. 2023; Zelle et al. 2010; Zhou et al. 2002). The usage of NH_3_ and NH_4_OH is frequently reported in processes for lactic acid fermentation (Balakrishnan et al. 2020; Hetényi et al. 2011; Vaidya et al. 2005). However, studies researching malic acid production with *A. oryzae* avoid nitrogen‐based neutralizers since high C:N ratios of media are designed to promote nitrogen starvation (Ochsenreither et al. 2014). Knuf et al. reported transcriptional downregulation of TCA and upregulation of rTCA enzymes during nitrogen starvation, stimulating the NADH and ATP neutral accumulation of malic acid (Knuf et al. 2013). Similar stimulation of nitrogen limited conditions was reported for citric acid production with *Aspergillus foetidus* (Kristiansen and Sinclair 1979).

Regarding organic acid production with *A. oryzae*, reported processes differ widely in pH regulation. Some processes are conducted at strongly acidic pH due to a lack of pH control or at neutral pH supported by CaCO_3_ or phosphate buffers (Brown et al. 2013; Ogawa et al. 1995; Raksha et al. 2012). Others neutralize at alkaline pH using MgCO_3_ (Brink et al. 2022). Nevertheless, the pH‐ and neutralizer‐dependent nature of organic acid production of *A. oryzae* remains to be explored.

To the best of our knowledge, this study offers the first comprehensive evaluation of organic acid production with *A. oryzae* concerning the utilization of hydroxides and carbonates as neutralizing agents as well as the pH spectrum across cultivation scales. These insights contribute to a deeper understanding and optimization of previously published studies and support the development of novel production processes.

## 2 Methods

### 2.1 Microorganism and Media

*A. oryzae* DSM 1863 was obtained from DSMZ strain collection (Deutsche Sammlung von Mikroorganismen und Zellkulturen GmbH, Braunschweig, Germany). All media were prepared using demineralized water. For conidia propagation, most of the protocol of Kövilein et al. was adapted (Kövilein et al. 2021). The fungus was grown on *Aspergillus* minimal medium (Barratt et al. 1965; Song et al. 2001). The medium comprised 15 g L^−1^ glucose monohydrate, 6 g L^−1^ NaNO_3_, 22.37 g L^−1^ KCl, 0.52 g L^−1^ MgSO_4_·7H_2_O, 1.52 g L^−1^ KH_2_PO_4_, 15 g L^−1^ agar and 2 mL L^−1^ of Hutner's Trace Elements (HTE). The pH was adjusted to 6.5 using NaOH, the medium was autoclaved for 20 min at 121°C. HTE solution consisted of 5 g L^−1^ FeSO_4_·7H_2_O, 50 g L^−1^ Na_2_EDTA, 22 g L^−1^ ZnSO_4_·7H_2_O, 11 g L^−1^ H_3_BO_3_, 5 g L^−1^ MnCl_2_·4H_2_O, 1.6 g L^−1^ CoCl_2_·6H_2_O, 1.6 g L^−1^ CuSO_4_·5H_2_O and 1.1 g L^−1^ (NH_4_)_6_Mo_7_O_24_·4H_2_O at pH 6.5 and was sterilized by filtration with a pore diameter of 0.2 µm (Hill and Kafer 2001). After incubation on agar plates for 6 days at 30°C, conidia were harvested in 50% (vol/vol) glycerol, filtered with Miracloth (Merck KGaA, Darmstadt, Germany), counted and stored in aliquots at ‐ 20°C.

The composition of preculture and main culture media followed the methodology outlined by Kövilein et al. (Kövilein et al. 2022). The preculture medium consisted of 40 g L^−1^ glucose monohydrate, 4 g L^−1^ (NH_4_)_2_SO_4_, 0.75 g L^−1^ KH_2_PO_4_, 0.98 g L^−1^ K_2_HPO_4_, 0.1 g L^−1^ MgSO_4_·7H_2_O, 0.1 g L^−1^ CaCl_2_·2H_2_O, 5 mg L^−1^ NaCl and 5 mg L^−1^ FeSO_4_·7H_2_O. All components of the preculture medium, except for HTE, were autoclaved together. Before inoculation, 2 mL L^−1^ of HTE was added to the preculture medium. The main culture medium was composed of 120 g L^−1^ glucose monohydrate, 1.2 g L^−1^ (NH_4_)_2_SO_4_, 0.1 g L^−1^ KH_2_PO_4_, 0.17 g L^−1^ K_2_HPO_4_, 0.1 g L^−1^ MgSO_4_·7H_2_O, 0.1 g L^−1^ CaCl_2_·2H_2_O, 5 mg L^−1^ NaCl, and 60 mg L^−1^ FeSO_4_·7H_2_O. Crude FeSO_4_·7H_2_O was used for precultures, while FeSO_4_·7H_2_O in 0.1 M H_2_SO_4_ was used for main cultures. For the main culture medium, KH_2_PO_4_, K_2_HPO_4_, NaCl, and (NH_4_)_2_SO_4_ were autoclaved together, with 100 µL of antifoaming agent Contraspum A 4050 HAc (Zschimmer & Schwarz GmbH, Lahnstein, Germany) added for stirred tank reactor (STR) cultivations. For one STR cultivation, 90 g L^−1^ CaCO_3_ buffer was autoclaved together with the previously mentioned salts. Glucose monohydrate was autoclaved separately, while FeSO_4_·7H_2_O, CaCl_2_·2H_2_O, and MgSO_4_·7H_2_O were sterilized by filtration. In the shake flask (SF) experiment, crude Mg_5_(CO_3_)_4_(OH)_2_·5H_2_O was autoclaved in SF separately.

### 2.2 Preculture Conditions

In 500 mL baffled SF 100 mL of preculture medium with 2 × 10^5^ conidia mL^−1^ was incubated at 30°C, 100 rpm in an orbit diameter of 25 mm for 24 h. The biomass was filtered with Miracloth and washed with demineralized water. The main cultures in SF and STR were inoculated with 7.5 g L^−1^ washed biomass (Kövilein et al. 2022).

### 2.3 Main Culture for Organic Acid Production in Shake Flasks

In the SF experiment, 100 mL inoculated main culture medium was cultivated in 500 mL baffled SF at 32°C, 120 rpm in an orbit diameter of 25 mm for 10 days. For sampling, 3 mL biomass‐free broth was collected. All SF cultivations were performed as biological quadruplicates.

### 2.4 Main Culture for Organic Acid Production in Stirred Tank Reactors

In STR cultivations, 1.4 L main culture was cultivated in 2.5 L bioreactors (Minifors, Infors AG, Bottmingen, Switzerland) at 32°C, 400 rpm with two Rushton turbines with diameters of 4.5 cm and 0.7 nL min^‐1^ ambient air for 10 d. For cultivations with different neutralizers, the pH was adjusted using 4 M NaOH, 2 M Na_2_CO_3_, 4 M KOH, 2 M Mg(OH)_2_, 2 M Ca(OH)_2_, or 2 M CaCO_3_ in combination with 4 M H_3_PO_4_ at pH 6.50 ± 0.05. To avoid particle settlement, Mg(OH)_2_, Ca(OH)_2_ and CaCO_3_ suspensions were continuously stirred during the cultivation. In one 4 M NaOH neutralized cultivation, 5% CO_2_‐enriched air was used for aeration (Zelle et al. 2010). For cultivations with variable pH, the pH was adjusted using 4 M NaOH for pH 6.50 ± 0.05 and above, 3 M for pH 6.00, 2 M for pH 5.50 and 1 M for pH 4.50 in combination with 4 M H_3_PO_4_. Neutralizers were stored on balances during cultivation; hence, the correlation of gravimetrically measured mass of consumed neutralizers, their densities and the time‐dependent pumping rate were used to calculate the consumed volume of neutralizers. For comparison between experiments, the consumed volume of neutralizer was divided by the initial cultivation volume $V_{0}$ of 1.4 L. An overview of carboxylic groups yielded from added alkali equivalents under varying neutralizer and pH conditions is provided in Supplement 3. The pH probe (EasyFerm Plus, Hamilton Bonaduz AG, Bonaduz, Switzerland) was two‐point calibrated with either pH 4.00 and 7.00 or pH 7.00 and 9.00, depending on the experiment. The dissolved oxygen tension (DOT) sensor (VisiFerm, Hamilton Bonaduz AG, Bonaduz, Switzerland) was two‐point calibrated with 100% nitrogen and 100% air. The off‐gas analytics (BlueVary, BlueSens gas sensor GmbH, Herten, Germany) for measurement of absolute humidity and partial pressures of O_2_ and CO_2_ were one‐point calibrated with ambient air. To prevent mycelial attachments on interior walls, reactor baffles were removed. To prevent mycelial attachments on the DOT sensor, convex‐designed sensor caps (ODO cap H2, Hamilton Bonaduz AG, Bonaduz, Switzerland) were used and the lower impeller was elevated 1 cm above the lower end of the stirring shaft with a distance of 6.5 cm to the higher impeller. A curved metal tube with a single outlet was used for aeration to minimize the risk of sparger clogging. For sampling, 10 mL biomass‐containing broth was removed from the reactors. All STR cultivations were performed as biological duplicates.

### 2.5 Analytics

Samples of the SF experiment were measured for pH and electrical conductivity with a conductometer (G1410, Senseca Germany GmbH, Regenstauf, Germany). The samples were stored at ‐ 20°C for metabolite analysis. All samples of STR experiments were sieved with 0.5 mm mesh size, the solid residue was washed with demineralized water, scanned at 2400 dpi (Perfection V600 Photo, Epson Deutschland GmbH, Düsseldorf, Germany), dried at 80°C for at least 2 days, weighted and annotated as cell dry weight (CDW) (Ferreira et al. 2014; Uwineza et al. 2021). The same procedure was performed for the last sample of the SF experiment. Values of CDW are contained in Supplement 1, biomass scans are provided in Supporting Information S2 and S4. The filtrate was stored at ‐ 20°C for metabolite analysis. The biomass of one reactor duplicate was repeatedly washed, flash‐frozen in liquid nitrogen, pulverized using a mortar, freeze‐dried and sent to the Fraunhofer Institute for Interfacial Engineering and Biotechnology IGB (Stuttgart, Germany) for elemental quantification.

Ammonium concentrations, as shown in Supporting Information S1, were determined photometrically using the Spectroquant assay kit (114752, Merck KGaA, Darmstadt, Germany). The assay was miniaturized to a 200 µL volume and filtrate from each reactor was measured in singlicate in microtiter plates following the manufacturer's instructions (Kövilein et al. 2022).

For metabolite quantification, methods from Kövilein et al. were adapted (Kövilein et al. 2021). Cultivation broth of SF and filtrate of STR cultivations were diluted 10‐fold with 0.66 M H_2_SO_4_, incubated at 80°C, 1000 rpm in an orbit diameter of 3 mm for 20 min and centrifuged for 5 min at 17,000*g*. The supernatant was analyzed by HPLC (Agilent 1100 Series, Agilent Technologies, Santa Clara, California, United States) equipped with a Rezex ROA organic acid H+ (8%) column (300 × 7.8 mm) and a Rezex ROA organic acid H+ (8%) guard column (Phenomenex, Aschaffenburg, Germany). Isocratic separation was performed using an injection volume of 10 µL, 5 mM H_2_SO_4_ at 0.5 mL min^‐1^ as eluent and a separation temperature of 30°C. Glucose was detected with a refractive index detector, organic acids were detected with a UV detector at 220 nm.

### 2.6 Calculations

Calculations for yields, productivities, gas transfer rates, molar ratios, consumed carbon fractions and yields of carboxylic groups were performed using Microsoft Excel (Version 2108, Microsoft Corporation, Redmond, Washington, United States). OriginPro (Version 2023, OriginLab Corporation, Northampton, Massachusetts, United States) was used for metabolite mass analysis, volume balancing and statistical analysis.

To compare performance of STR cultivations without dilution effects, masses of metabolites $m_{M}\left(t\right)$ at fermentation time t were calculated with the metabolite concentration $c_{M}\left(t\right)$ in the culture broth, the cultivation volume V(t) and the sum of previously sampled metabolites $\sum_{t_{i}\;\leq t}c_{M}(t_{i})V_{s,i}$, as shown in Equation 1 (Battling et al. 2022). Metabolite concentrations in STR cultivations were calculated as mass per initial cultivation volume $V_{0}$. In contrast, metabolite concentrations in SF cultivations correspond to concentrations present in the culture broth.

(1) $$m_{M}\left(t\right)=c_{M}\left(t\right)V(t)+\sum_{t_{i}\;\leq t}c_{M}(t_{i})V_{s,i}.$$

The cultivation volume V(t) was calculated by Equation 2, with the added neutralizer volume $V_{n}(t)$, the sampled volume $V_{s}(t)$ and evaporation volume $V_{e}(t)$. Except for the cultivation neutralized with Ca(OH)_2_, volume balances only consider base addition as neutralizer, since the minimal amounts of added H_3_PO_4_ proved to be gravimetrically nonquantifiable.

(2) $$V(t)=V_{0}+V_{n}(t)-V_{s}(t)-V_{e}(t).$$

Evaporation was calculated by Equation 3, with the sum of the evaporated volume during the measuring intervals of the off‐gas analyzer ${\Delta t}_{m}$, the molar gas volume flow $\frac{p_{\text{gas}}F_{\text{gas},\text{in}}}{RT_{\text{gas}}}$, the absolute humidity in the off‐gas $f_{\text{off}}(t)$, the molar mass $M_{w}$ and the density of water $\rho_{w}$ (Mueller and Arto 2025). For all cultivations, in‐gas humidity $f_{\text{in}}$ was estimated to 0.1%, calculated as the average of dry gas measurements taken over several hours.

(3) $$V_{e}(t)=\sum_{t_{0}}^{t}\left(\frac{p_{\text{gas}}F_{\text{gas},\text{in}}{\Delta t}_{m}}{RT_{\text{gas}}}\;\frac{{(f}_{\text{off}}(t)-f_{\text{in}})M_{w}}{\rho_{w}}\right).$$

Oxygen transfer rate (OTR, O_2_), carbon transfer rate (CTR, CO_2_) and respiratory quotient (RQ) were calculated following Equations 4, 5, and 6 using the O_2_ and CO_2_ fraction of the in‐ and off‐gas. The off‐gas flow was calculated assuming inert nitrogen. For comparability, gas transfer rates were calculated as rate per initial cultivation volume $V_{0}$.

(4) $$\text{OTR}\;=\frac{p_{\text{gas}}F_{\text{gas},\text{in}}}{RT_{\text{gas}}}\left(y_{O_{2},\text{in}}-\frac{1-y_{O_{2},\text{in}}-y_{{\text{CO}}_{2},\text{in}}}{1-y_{O_{2},\text{off}}-y_{O_{2},\text{off}}}y_{O_{2},\text{off}}\right).$$

(5) $$\text{CTR}\;=\frac{p_{\text{gas}}F_{\text{gas},\text{in}}}{RT_{\text{gas}}}\left(y_{{\text{CO}}_{2},\text{ex}}\frac{1-y_{O_{2},\text{in}}-y_{{\text{CO}}_{2},\text{in}}}{1-y_{O_{2},\text{off}}-y_{O_{2},\text{off}}}-y_{{\text{CO}}_{2},\text{in}}\right).$$

(6) $$\text{RQ}\;=\frac{\text{CTR}}{\text{OTR}}.$$

Molar ratios of organic acids $R_{\text{OA},i}$ were calculated following Equation 7, dividing the molar amount of a produced organic acid $n_{\text{OA},i}$ by the total amount of produced organic acids $\sum n_{\text{OA}}$.

(7) $$R_{\text{OA},i}=\frac{n_{\text{OA},i}}{\sum n_{\text{OA}}}.$$

Consumed carbon fractions from glucose $F_{M,i}$ were calculated following Equation 8, dividing the carbon content of a produced metabolite $n_{\text{c in M},i}$ by the amount of consumed carbon from glucose $n_{\text{c from Glc}}$.

(8) $$F_{M,i}=\frac{n_{\text{c in M},i}}{n_{\text{c from Glc}}}.$$

Statistical significance was assessed using one‐way ANOVA followed by Tukey's post‐hoc test to evaluate differences in organic acid yields obtained using NaOH, NaOH with 5% CO_2_ and Na_2_CO_3_ as neutralizing conditions. Differences were considered weakly significant at *p* < 0.05, significant at *p* < 0.01 and strongly significant at *p* < 0.001.

## 3 Results

### 3.1 Organic Acid Production With Different Neutralizers

An excess of CaCO_3_ buffer is used as a reference neutralizer in organic acid production, while pH‐controlled addition of CaCO_3_ is applied to evaluate the effects of oversaturation in contrast to demand‐based supplementation. Hydroxides of K^+^, Na^+^, Ca^2+^, and Mg^2+^ are used to analyze the influence of different metal ion identities in neutralizers. Na_2_CO_3_ is included to isolate the impact of carbonate addition. Due to reports of increased malic and succinic acid yields in *Saccharomyces cerevisiae* by Zelle et al. with 5% CO_2_‐enriched air, one duplicate was aerated with the same enrichment (Zelle et al. 2010).

**Figure 1:**
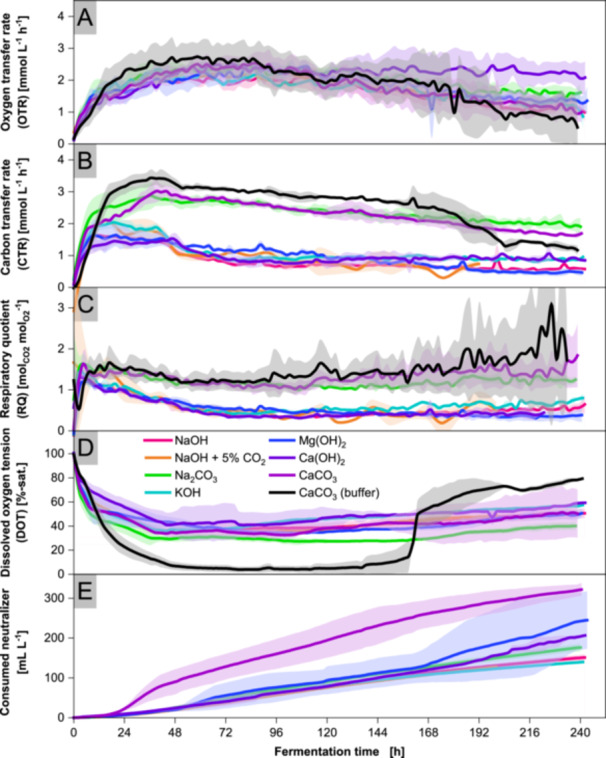
Online monitoring of cultivations with *Aspergillus oryzae* DSM 1863 in 2.5 L STR with different neutralizers. Cultivations were performed with X_0_ of 7.5 g L^−1^ biomass, 109 g L^−1^ glucose, V_0_ of 1.4 L, 32°C, 400 rpm and 0.7 nL min^−1^ air. pH was maintained at 6.50 ± 0.05 with 4 M NaOH (+ 5% CO_2_), 2 M Na_2_CO_3_, 4 M KOH, 2 M Mg(OH)_2_, 2 M Ca(OH)_2_, 2 M CaCO_3_ and 4 M H_3_PO_4_ or 90 g L^−1^ CaCO_3_ as buffer. CDW, pH of CaCO_3_ buffered cultivations, biomass scans and yield of carboxylic groups are contained in Supporting Information S1 to S3. The online signals were averaged over 100 data points. Means are represented by solid lines, while deviations are shown as shaded areas of the same color. Data represent means ± standard deviations of biological duplicates.

Figure 1 compares alkali and alkaline earth hydroxides and carbonates in terms of microbial growth patterns. OTR and CTR provide information on the metabolic activity of the fungus during cultivation. After inoculation, OTR and CTR increase rapidly. The CTR of hydroxide neutralized cultures form a plateau which decreases after 2 days. A similar pattern is observed for the cultures with carbonate neutralizing agents, although the CTR reaches values twice as high as those of the hydroxide‐based neutralizing cultures. In general, higher CTR values for carbonate‐based neutralizers are attributed to the acid‐carbonate reaction, releasing CO_2_. Cultivations buffered with CaCO_3_ maintain a pH of 6.80, shown in Supporting Information S1. The DOT declines to single digits and rapidly increases due to air entrainment by the upper impeller following a drop in liquid level from evaporation. The low RQ values in hydroxide‐neutralized cultivations reflect the formation of organic acids, whose higher oxidation state relative to the substrate typically results in RQ values below 1. The high CDW observed in cultivations neutralized with dosed CaCO_3_ and buffering CaCO_3_ are attributed to the accumulation of water‐insoluble particles in the fungal biomass, as evidenced by the 2D biomass images shown in Supporting Information S2. A similar phenomenon is observed during the last days of cultivation neutralized with Ca(OH)_2_.

**Figure 2:**
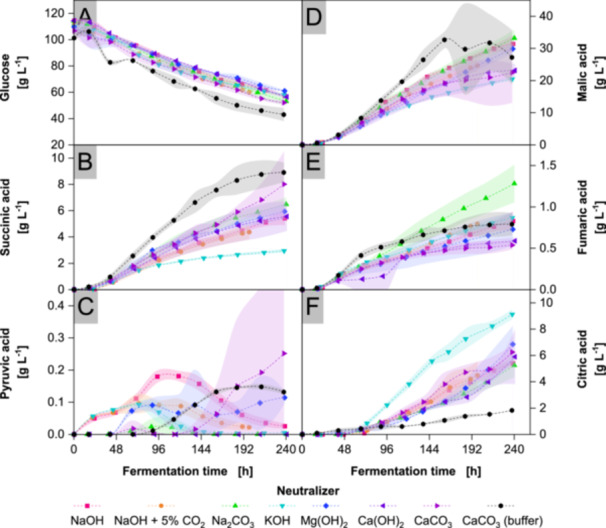
Metabolites in cultivations with *Aspergillus oryzae* DSM 1863 in 2.5 L STR with different neutralizers. Cultivations were performed with X_0_ of 7.5 g L^−1^ biomass, 109 g L^−1^ glucose, V_0_ of 1.4 L, 32°C, 400 rpm and 0.7 nL min^−1^ air. pH was maintained at 6.50 ± 0.05 with 4 M NaOH (+ 5% CO_2_), 2 M Na_2_CO_3_, 4 M KOH, 2 M Mg(OH)_2_, 2 M Ca(OH)_2_, 2 M CaCO_3_ and 4 M H_3_PO_4_ or 90 g L^−1^ CaCO_3_ as buffer. CDW, pH of CaCO_3_ buffered cultivations, biomass scans and yield of carboxylic groups are contained in Supporting Information S1 to S3. Data represent means ± standard deviations of biological duplicates. Dashed lines and Akima‐spline‐connected standard deviations are provided for visual guidance.

The choice of neutralizer, as shown in Figure 2, impacts the overall glucose consumption, which ranges from 49 g L^−1^ with Mg(OH)_2_ to 61 g L^−1^ with Na_2_CO_3_. Na^+^ and Mg^2+^‐based neutralizers produce higher concentrations of malic and fumaric acids than K^+^ and Ca^2+^‐based neutralizers, as shown in Table 1. The use of KOH results in the lowest final concentrations of malic and succinic acids, while stimulating citric acid production. The organic acid profile with Mg(OH)_2_ appears similar to that of Na^+^‐based neutralizing agents. The organic acid profiles of dosed Ca(OH)_2_ and CaCO_3_ align for citric, fumaric and malic acid while dosed CaCO_3_ shows higher productivity for succinic and pyruvic acid. Buffering with CaCO_3_ yields substantially lower citric acid productivity than any other agent. In addition, buffering with CaCO_3_ shows highest productivity for succinic and malic acid until 161 h. For pyruvic acid, a general trend of secretion and reuptake is observed. As shown in Table 2, the yields of succinic and malic acid vary across the Na^+^‐based neutralizers, with statistically weak significance observed only for the malic acid yield between NaOH + 5% CO_2_ and Na_2_CO_3_.

**Table 1:** Key performance parameters of dosed neutralizers for production of most abundant organic acids with *Aspergillus oryzae* DSM 1863. Performance was oriented on the highest productivity yielded with the specific neutralizer. Averaged productivities and yields were calculated based on endpoint measurements.

Organic acid	Best performing (dosed) neutralizer	c_max_ [g L^−1^]	Y_OA/S_ [mg g^−1^]	P_OA_ [mg L^−1^ h^−1^]	P_OA_ [mM h^−1^]
Malic	Na_2_CO_3_	33.18 ± 2.59	544 ± 11	138 ± 11	1.03 ± 0.08
Citric	KOH	9.12 ± 0.39	176 ± 3	38 ± 2	0.20 ± 0.01
Succinic	CaCO_3_	8.02 ± 2.49	146 ± 42	34 ± 10	0.29 ± 0.09
Fumaric	Na_2_CO_3_	1.28 ± 0.23	21 ± 3	5 ± 1	0.05 ± 0.01

**Table 2:** Yields of Na^+^‐based neutralizers for production of malic and succinic acid with *Aspergillus oryzae* DSM 1863. Calculations are based on sampling after 190 h. Statistical significance of yield differences are only displayed for *p*‐values equal or smaller than 0.05.

Organic acid	Y_OA/S_ [mg g^−1^]		
	NaOH	NaOH + 5% CO_2_	Na_2_CO_3_
Succinic	79 ± 3	87 ± 8	90 ± 2
Malic	470 ± 15	488 ± 18^a^	425 ± 5^a^

^a^ *p* = 0.041 is considered weakly significant.

**Figure 3:**
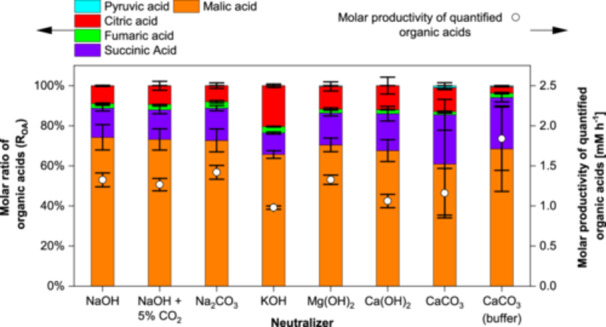
Molar ratio and molar productivity of quantified organic acids in cultivations with *Aspergillus oryzae* DSM 1863 in 2.5 L STR with different neutralizers. Ratios and productivities were calculated for endpoint measurements. For CaCO_3_ buffered cultivations, measurements at 161 h before rise of DOT are considered. Cultivations were performed with X_0_ of 7.5 g L^−1^ biomass, 109 g L^−1^ glucose, V_0_ of 1.4 L, 32°C, 400 rpm and 0.7 nL min^−1^ air. pH was maintained at 6.50 ± 0.05 with 4 M NaOH (+ 5% CO_2_), 2 M Na_2_CO_3_, 4 M KOH, 2 M Mg(OH)_2_, 2 M Ca(OH)_2_, 2 M CaCO_3_ and 4 M H_3_PO_4_ or 90 g L^−1^ CaCO_3_ as buffer. Data represent means ± standard deviations of biological duplicates.

When comparing the total molar productivities in Figure 3, buffering with CaCO_3_ exhibits the highest total molar productivity of acids. Among the dosed neutralizers, Na^+^ and Mg^2+^ result in the highest productivities. In contrast, the lowest overall productivity is observed with KOH. Among organic acids, malic acid shows the highest molar ratio of organic acids at 75% buffering with CaCO_3_ while dosed CaCO_3_ produces 58%. Highest ratios for succinic acid are yielded with dosed CaCO_3_ while KOH produces the lowest ratio with 10%. In contrast, KOH produces the highest ratio of citric acid while buffering with CaCO_3_ yields the lowest ratio with 2%.

### 3.2 Organic Acid Production at Different Controlled pH Levels

NaOH was selected to control pH levels due to its water solubility, high productivity for most organic acids and lack of interference with microbial CO_2_ emissions. The different molar concentrations of NaOH were selected to ensure stable pH regulation even at acidic conditions.

**Figure 4:**
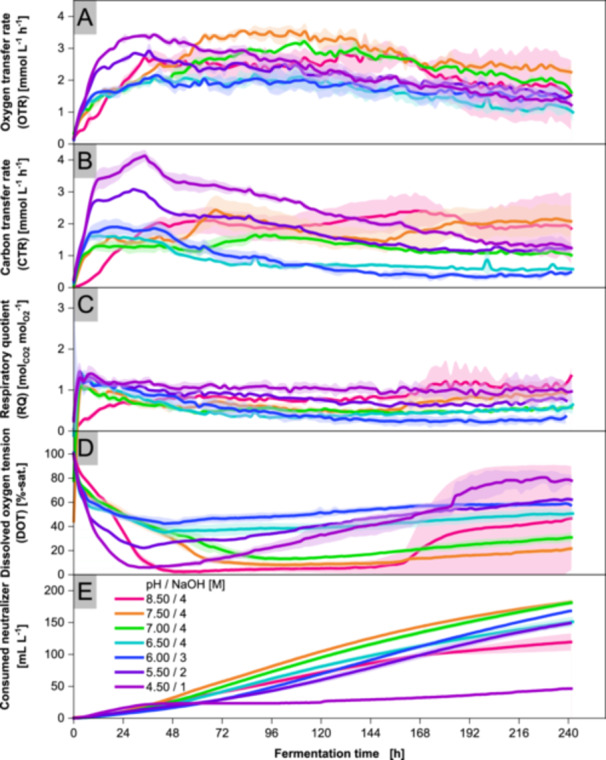
Online monitoring of cultivations with *Aspergillus oryzae* DSM 1863 in 2.5 L STR at varying pH. Cultivations were performed with X_0_ of 7.5 g L^−1^ biomass, 109 g L^−1^ glucose, V_0_ of 1.4 L, 32°C, 400 rpm and 0.7 nL min^−1^ air. pH was maintained within ± 0.05 with NaOH and 4 M H_3_PO_4_. Ammonium concentrations, CDW, biomass scans and yield of carboxylic groups are contained in Supporting Information S1 to S3. The online signals were averaged over 100 data points. Means are represented by solid lines, while deviations are shown as shaded areas of the same color. Data represent means ± standard deviations of biological duplicates.

Figure 4 shows that the timing of the CTR decline remains unchanged across pH levels from 4.50 to 6.50. However, both the levels of the CTR and OTR plateaus increase as pH decreases, with the culture at pH 4.50 reaching more than twice the CTR of the culture at pH 6.50. In contrast, the maximum CDW at pH 4.50 is only about 25% higher than at pH 6.50. Cultivations at pH 7.00 and 7.50 display CTR plateaus that transition into increases following ammonium depletion. At pH 8.50, the respiratory activity increases considerably more slowly during the initial hours compared to the other pH conditions.

**Figure 5:**
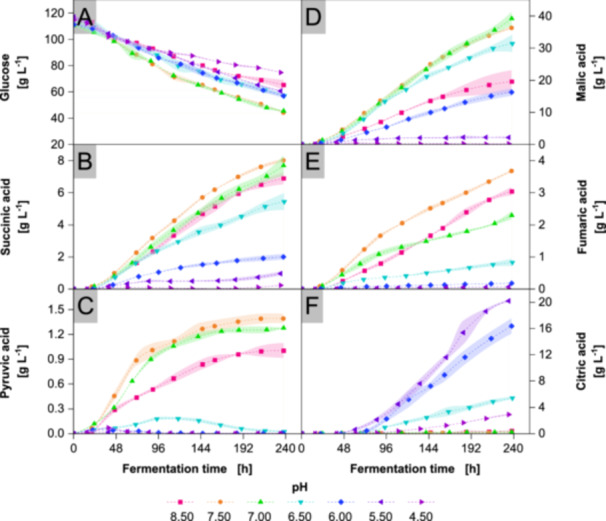
Metabolites in cultivations with *Aspergillus oryzae* DSM 1863 in 2.5 L STR at constant pH. Cultivations were performed with X_0_ of 7.5 g L^−1^ biomass, 109 g L^−1^ glucose, V_0_ of 1.4 L, 32°C, 400 rpm and 0.7 nL min^−1^ air. pH was maintained within ± 0.05 with NaOH and 4 M H_3_PO_4_. Ammonium concentrations, CDW, biomass scans and yield of carboxylic groups are contained in Supporting Information S1 to S3. Data represent means ± standard deviations of biological duplicates. Dashed lines and Akima‐spline‐connected standard deviations are provided for visual guidance.

Figure 5 shows slower glucose consumption at highly acidic or alkaline pH levels compared to neutral pH. Acidic conditions lead to notably lower productivities for malic, succinic, fumaric, and pyruvic acids compared to higher pH levels. Interestingly, at acidic pH, *A. oryzae* produces high amounts of citric acid, reaching up to 20 g L^−1^. In contrast, neutral pH stimulates the production of the other acids, with pH 7.00 yielding the highest concentration of malic acid, while pH 7.50 results in higher levels of succinic, fumaric, and pyruvic acids, as shown in Table 3. The cycle of pyruvic acid secretion and reuptake occurs at all tested pH values at or below 6.50, but is most evident at pH 6.50 due to higher secretion levels. Especially for neutral pH, the first 3 d yield 65% of pyruvic acid totally produced.

**Table 3:** Key performance parameters of pH levels in production of most abundant organic acids with *Aspergillus oryzae* DSM 1863. Performance was oriented on the highest productivity yielded with the controlled pH level. Averaged productivities and yields were calculated based on endpoint measurements.

Organic acid	Best performing pH level	c_max_ [g L^−1^]	Y_OA/S_ [mg g^−1^]	P_OA_ [mg L^−1^ h^−1^]	P_OA_ [mM h^−1^]
Malic	7.00	39.14 ± 2.13	601 ± 25	165 ± 9	1.23 ± 0.07
Citric	5.50	20.18 ± 0.21	359 ± 8	86 ± 1	0.45 ± 0.01
Succinic	7.50	8.00 ± 0.10	114 ± 1	34 ± 1	0.29 ± 0.01
Fumaric	7.00	2.29 ± 0.13	35 ± 2	10 ± 1	0.08 ± 0.01

Most cultivations achieve CDW between 3 and 4 g L^−1^. After cultivation, freeze‐dried biomass was subjected to elemental analysis: C content of 44.5 ± 0.5%, H of 7.1 ± 0.1% and N of 3.9 ± 0.2% were determined, respectively. Assuming total conversion of measured ammonium into biomass, theoretical maxima of CDW between 5.2 g L^−1^ and 6.4 g L^−1^, respectively in cultivations at pH 7.00 and 4.50, could have been reached. The measured CDW indicates that about three quarters of nitrogen from ammonium is converted into biomass during the initial growth phase. The reduced yield may result from the secretion of nitrogenous compounds (Liu et al. 2014).

**Figure 6:**
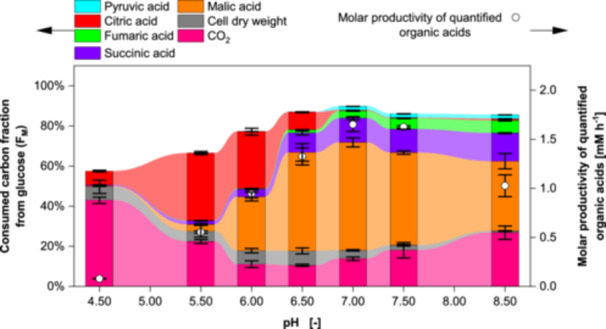
Consumed carbon fraction from glucose and molar productivity of quantified organic acids in cultivations with *Aspergillus oryzae* DSM 1863 in 2.5 L STR at varying pH. Fractions and productivities were calculated for endpoint measurements. Cultivations were performed with X_0_ of 7.5 g L^−1^ biomass, 109 g L^−1^ glucose, V_0_ of 1.4 L, 32°C, 400 rpm and 0.7 nL min^−1^ air. pH was maintained within ± 0.05 with NaOH and 4 M H_3_PO_4_. Data represent means ± standard deviations of biological duplicates. Connected bars are provided for visual guidance.

The total productivity of organic acids peaks at neutral pH, as shown in Figure 6. Alkaline and acidic pH reduce the productivity, also reflected by the base consumption. In principle, the base consumption shows a correlation with acid production and may serve as an indicator of productivity in future processes. At acidic pH, the largest fraction of carbon from glucose is consumed for CO_2_ and citric acid production, while with increasing pH this share declines in favor of malic, succinic and fumaric acid. At alkaline pH, the fraction of carbon consumed for malic acid declines along the increase of the carbon share consumed for CO_2_.

### 3.3 Organic Acid Production at Shifting pH Levels

In the previous experiments, the pH was maintained by demand‐based addition of neutralizers or by using an excess of buffer. In this experiment, different buffer concentrations were selected to investigate the effect of shifting pH on acid production. The buffer concentrations were deliberately kept low, so over the course of the fermentation the buffer capacities of 10 g L^−1^, 20 g L^−1^ and 30 g L^−1^ Mg_5_(CO_3_)_4_(OH)_2_·5H_2_O were expected to be depleted, leading to an acidic pH shift. This compound buffers at neutral to alkaline pH, providing a broader pH range compared to CaCO_3_.

**Figure 7:**
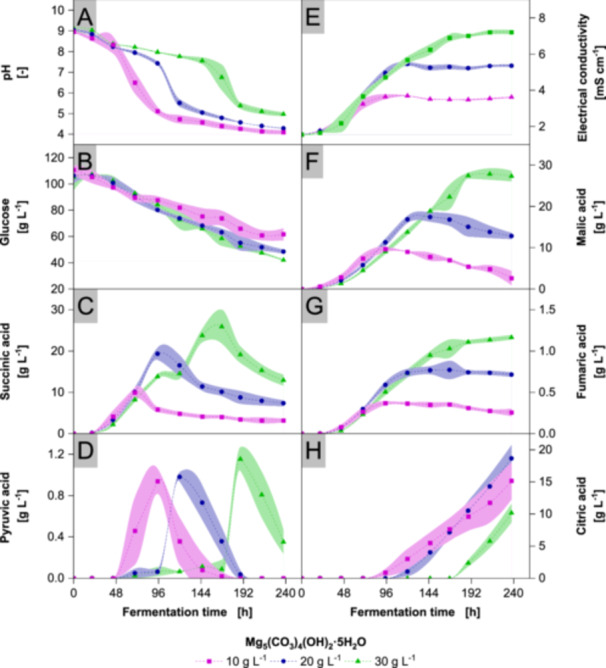
Metabolites in cultivations with *Aspergillus oryzae* DSM 1863 in 500 mL baffled SF with varying Mg_5_(CO_3_)_4_(OH)_2_·5H_2_O concentrations. Cultivations were performed with X_0_ of 7.5 g L^−1^ biomass, 109 g L^−1^ glucose, V_0_ of 100 mL, 32°C, 120 rpm in 25 mm. CDW, oxalic acid concentrations and biomass scans are contained in Supporting Information S1 and S4. Data represent means ± standard deviations of biological quadruplicates. Dashed lines and Akima‐spline‐connected standard deviations are provided for visual guidance.

During the initial phase shown in Figure 7, all three buffer concentrations maintain alkaline pH. After fermentation times of 69 h, 120 h, and 168 h, corresponding to buffer concentrations of 10 g L^−1^, 20 g L^−1^, and 30 g L^−1^ respectively, samples indicate a shift to acidic pH. The electrical conductivity correlates with ion concentration; therefore, released Mg^2+^ ions and anionic acids increase the electrical conductivity during buffered cultivations. Exemplary images of the flask, shown in Supporting Information S4, lack dispersed buffer, verifying the depletion of buffer capacity for all set of cultivations. Generally, all organic acids except citric acid are produced at alkaline to neutral pH. In all cultivations, most pyruvic acid is produced during the pH drop at neutral conditions. At acidic pH, pyruvic acid is consumed similar to the previously mentioned acids. The fungus initiates citric acid production only during the pH drop and, contrary to the other acids, maintains production during continuous acidification of the media. Interestingly, following the pH drop, the productivity of organic acids, except citric acid, markedly declines, with fumaric acid production almost ceasing entirely and concentrations of malic, succinic, and pyruvic acid even decreasing, suggesting microbial consumption.

## 4 Discussion

### 4.1 Organic Acid Production With Different Neutralizers

To investigate the influence of different neutralizers on growth and organic acid production, neutralizers from various alkali and alkaline earth metals were tested in cultivations at a controlled pH of 6.50. For all cultivations, the rapid increase in CTR and OTR indicates a metabolically highly active inoculum, even after washing with deionised water. The almost linear drop in DOT during the CTR plateau signals progressive, albeit limited, growth. Although the ratio of C:N in the medium is designed for nitrogen depletion, the cultivations develop the CTR plateau before ammonium depletion. In addition, nitrogen depletion would prohibit protein biosynthesis, leading to growth cessation. Accordingly, a so‐far unknown nutrient depletion must occur before ammonium depletion. The identification of this depleting nutrient lies beyond the scope of this study and is addressed in a separate study. The drop in CTR after the plateau correlates with the consumption of ammonium in the cultivations at different pH. Therefore, the drop in CTR can be used as an online measurement signal to indicate the onset of nitrogen limitation. The decrease in OTR in the second half of all cultures indicates a decrease in metabolic activity, as can be seen from the decelerating increase in acid concentrations, especially for succinic and malic acid. The activity decrease is most likely caused by the continued nitrogen depletion, recycling proteins to maintain cell activity (Gupta et al. 2024).

Up to the point of increasing DOT, the use of CaCO_3_ buffer resulted in the highest productivity of malic acid and the metabolically related succinic acid compared to the other neutralizers. Potential productivity‐enhancing effects include the release of Ca^2+^ ions, the elevated pH, the initially high particle concentration, the increased emission of CO_2_, the large proportion of loose mycelium and the low DOT. Ca^2+^ ions are unlikely to be the primary cause of the differences in acid production, as for dosed CaCO_3_ and Ca(OH)_2_ lower productivities are observed. A partial influence on the enhanced acid production can be linked to the elevated pH, shown in Supporting Information S1, as higher malic and succinic acid concentrations are produced with NaOH at pH 7.00, as shown in Figure 5. However, CaCO_3_ buffer outperforms NaOH at pH 7.00 in productivity until rapid rise in DOT, suggesting additional factors. For instance, the addition of water‐insoluble particles like CaCO_3_ to filamentous cultivations is referred to as MPEC (microparticle‐enhanced cultivation) and increases the productivity of various hydrolases of *A. oryzae* when cultivated with micro‐ and silver nanoparticles (Singh 2018). However, the impact of MPEC on organic acid production remains uncertain since, for example, *Rhizopus delemar* lacks changes in production of malic and fumaric acid with the addition of plaster sand at concentrations up to 120 g L^−1^ (Ronoh et al. 2022). Further, the productivity‐enhancing effect for malic acid by buffering with CaCO_3_ cannot be attributed solely to carbonate release, as malic acid production remains similar between NaOH, NaOH + 5% CO_2_ and Na_2_CO_3_, as well as between dosed Ca(OH)_2_ and CaCO_3_. However, the increased formation of fumaric and succinic acid with Na_2_CO_3_ indicates a shift in metabolic flux rather than a direct stimulation of malic acid synthesis. Another stimulating effect in buffering with CaCO_3_ could be caused by the morphological phenotype. Densely packed mycelium in the form of pellets is considered less productive compared to loose mycelium, as diffusion limitations hinder nutrient supply to the pellet core regions (Di Lorenzo et al. 2022). For instance, *R. delemar* exhibits higher fumaric acid productivity with smaller pellets (Zhou et al. 2011). In this study, the high proportion of free mycelium, as shown in Supporting Information S2, may contribute to increased productivity by enhancing general nutrient uptake.

The low DOT caused by the buffer CaCO_3_ indicates oxygen limitation, which typically needs to be avoided in aerobic microorganisms. However, studies have shown that hypoxia stimulates accumulation of certain organic acids (Meijer et al. 2007). *A. oryzae* upregulates genes for the glyoxylate pathway during hypoxia, increasing the conversion of isocitric to malic acid and bypassing NADH accumulation in the oxidative TCA cycle (Terabayashi et al. 2012). Conversion of isocitric acid through the glyoxylate pathway could cause the observed low citric acid productivity during hypoxia. The hypothesis of hypoxia‐related modulation of organic acid production is further supported by the simultaneous rise in DOT and collapse in malic and succinic acid productivity in CaCO_3_ buffered cultivations as well as by generally lower malic acid productivity observed during normoxia with dosed neutralizers. An excess of water‐insoluble CaCO_3_ buffer could reduce oxygen availability by impeding mass transfer, as shown for silica gel and sand particles (Pereira et al. 2024). For *Aspergillus niger*, Diano et al. report similar hypoxia‐related effects and supposes stimulated malic acid production as necessary reoxidation of NADH during hypoxia (Diano et al. 2009).

Furthermore, differences in acid production are observed depending on the identity of the metal ions. In the present study, KOH generates the lowest productivity for succinic and malic acid, while its usage shows highest productivity for citric acid compared to other neutralizers. Similar phenomena are observed in *Aureobasidium pullulans*, with KOH reducing poly malic acid production by 50% compared to NaOH, in *Corynebacterium crenatum* reducing succinic acid production by 25%, in *Mannheimia succinicproducens* reducing succinic acid production by 20%, in *E. coli* reducing succinic acid production by 20%, in *Sporolactobacillus inulinus* reducing lactic acid production by 15% and in *Sporolactobacillus nakayamae* reducing lactic acid production by 8% compared to NaOH (Andersson et al. 2009; Cao et al. 2020; Chen et al. 2017; Kim et al. 2024; Michelz Beitel et al. 2017; Wu et al. 2019). Even in nonacid production processes similar observations are reported. For instance, *Klebsiella oxytoca* produces 25% less 2,3‐butanediol when KOH is used instead of NaOH (Cho et al. 2015). K^+^ is the most abundant alkali metal in the cytoplasm, contributing to cytoplasmic charge balance, turgor pressure and other metabolic functions (Clarkson and Hanson 1980; Epstein and Schultz 1965). In fungi, the H^+^‐ATPase expels protons, creating a membrane potential and proton gradient (Serrano 1984). This electrochemical gradient powers TKR type transporters, enabling K^+^ accumulation via secondary active transport mechanisms (Corratgé‐Faillie et al. 2010). High extracellular K^+^ concentrations may influence cellular metabolism by altering ion homeostasis, which, among other effects, could impact the production and secretion of organic acids.

Regardless of whether NaOH or KOH is used, the resulting Na^+^‐ or K^+^‐salts of organic acids remain highly soluble, preventing precipitation and eliminating recovery routes commonly used in Ca^2+^‐based systems. Once precipitation is no longer feasible, a range of recovery options must be considered, each with its own technical implications and potential economic considerations. Huang et al. present electrodialysis as a promising membrane‐based alternative in organic acid recovery, highlighting its potential to overcome limitations associated with conventional methods such as crystallization and solvent extraction, particularly regarding environmental impact and process efficiency (Huang et al. 2007). K^+^ exhibits higher transport rates through ion‐exchange membranes, whereas Na^+^ is generally favored in industrial practice due to high availability of NaOH (Beaulieu et al. 2020; Jashni et al. 2019; Linares‐Solano et al. 2012). As shown in Supplement 3, this preference is further supported by higher yields of carboxylic groups from alkali equivalents observed for Na^+^‐based neutralizers compared to K^+^, Mg^2+^ and Ca^2+^ compounds, with particularly low yields for Mg^2+^ and Ca^2+^ likely resulting from low water solubilities. In addition, soluble alkalies such as NaOH and KOH could be regenerated via electrodialysis during organic acid recovery and reused as neutralizers in organic acid production (Wiśniewski et al. 2004).

While the choice of cation has clear implications for process performance, the nature of the alkaline anion, whether hydroxide or carbonate, also influences cellular metabolism. In general, different organisms show indications of increased activity in the rTCA branch upon usage of carbonate‐containing neutralizers. In fumaric acid production with *Rhizopus oryzae*, NaHCO_3_ and CaCO_3_ led to three‐ and four‐fold higher concentrations, respectively, compared to Ca(OH)_2_ (Zhou et al. 2002). Although the present study shows a less pronounced difference, higher fumaric acid concentrations are also achieved with Na_2_CO_3_ compared to NaOH. In *E. coli*, similar to our study, Na_2_CO_3_ increases succinic acid production by 10% in comparison to NaOH (Andersson et al. 2009). In contrast to our study, buffering with CaCO_3_ increases malic acid production in *Ustilago trichophora* even during normoxia, with the achieved concentration exceeding the production with NaOH by 40% (Zambanini et al. 2016). Also at normoxia, *A. pullulans* shows a one‐third lower polymalic acid production with NaOH compared to buffering with CaCO_3_ (Cao et al. 2020). Unlike the engineered *S. cerevisiae* strain reported by Zelle et al. our data show mostly insignificant differences in yields of malic and succinic acid across Na^+^‐based neutralizers, including NaOH, NaOH + 5% CO_2_ and Na_2_CO_3_. The discrepancy may result from insufficient CO_2_ dissolution, limiting carbonate formation during CO_2_‐enriched aeration or species‐specific preferences (Zelle et al. 2010). While our data primarily show carbonate‐based stimulation for succinic and fumaric acid production, all published evidence suggests stimulation of carbonate‐containing neutralizers on the activity of the rTCA branch is a cross‐species phenomenon.

### 4.2 Organic Acid Production at Different pH Levels

To investigate acid production at different controlled pH levels, STR cultivations were performed with pH control using NaOH, while SF cultures utilized a Mg^2+^‐based carbonate buffer with varying buffer capacities to induce dynamic pH shifts.

At neutral to alkaline pH, *A. oryzae* secretes mainly malic acid, succinic acid and fumaric acid. *R. delemar* also produces malic acid, but meaningful production occurs only at pH values above 6.50, with maximum concentrations reaching 10 g L^−1^ (Ronoh et al. 2022). In *P. ochrochloron*, neutral pH enhances the production of organic acids, while *U. trichophora* shows markedly higher malic acid yields at pH 6.50 compared to pH 5.50 or 4.50, aligning with observations from our study (Vrabl et al. 2012; Zambanini et al. 2016). Brink et al. cultivated *A. oryzae* in a fungal bed bioreactor and attributed its low malic acid yield of 3 g L^−1^ to missing Ca^2+^ from CaCO_3_ by using NaOH for neutralization (Brink et al. 2023). In light of our results, their low productivity could also be explained by the comparatively acidic pH of 6.00, at which, as we have shown, productivity drops by 60% compared to pH 7.00. In general, the cross‐species trend reveals that rTCA acids are produced at predominantly neutral pH.

It is important to consider that in this study organic acid production is analyzed without active regulation of DOT. Since reduced hypoxia in CaCO_3_ buffered cultivations likely enhances the production of rTCA‐associated acids, a similar effect at low DOT for cultivations at pH 7.00 and 7.50 is conceivable. Despite exhibiting the lowest DOT for most of the cultivation time, cultures at pH 8.50 produce fewer organic acids than those at neutral pH. Similarly, cultures at pH 4.50 and 5.50 show lower DOT than those at pH 6.00 but yield fewer organic acids. Accordingly, a potential influence of oxygen limitation appears to be partly overshadowed by pH effects. The specific impact of oxygen availability on acid production will be addressed in a future study.

Generally, cytosolic pH is essential for cell viability, affecting protein conformation and enzyme activity, driving ATP synthesis and regulating nutrient import (Kane 2016). In SF cultivations, the secreted organic acids dissociate at alkaline pH, decreasing the external pH of media while in some STR cultivations pH was initially lowered with H_3_PO_4_ and stabilized with NaOH at acidic pH. However, for fungi like *A. niger* and *Saccharomyces cerevisiae* neutral to slightly alkaline cytosolic pH values are reported (Hesse et al. 2002; Valli et al. 2006). Accordingly, organisms evolved strategies for cytosolic pH homeostasis and sensing of external pH. For example, if plasma membrane complexes of *Aspergillus nidulans* contact alkaline pH, they trigger the proteolytic activation of the pH‐response regulating transcription factor PacC (Peñas et al. 2007). As the primary barrier, the low permeable cytoplasmic membrane passively shields against uncontrolled proton diffusion (Hesse et al. 2002). Buffering systems absorb short‐term pH fluctuations while long‐term pH shifts are regulated by vacuolar and plasma membrane associated H^+^‐ATPases (Casey et al. 2010; Klionsky et al. 1990; Poznanski et al. 2013; Serrano 1991; Serrano et al. 1986). In addition, cation transporters support pH homeostasis by coupling H^+^ export with Na^+^ or K^+^ uptake (Brett et al. 2005; Sychrová et al. 1999). Hence, growth of *Ascomycetes* is reported for external pH as low as 2 (Liaud et al. 2014). *A. niger* maintains a pH gradient greater than 6 (Hesse et al. 2002). However, the majority of carboxylic groups protonate at pH below pKa, allowing diffusion of undissociated organic acids across cell membranes (Piper et al. 2001). At cytosolic pH, organic acids dissociate, the protons acidify the cytoplasm and affect growth by stressing diverse cellular activity as well as increasing ATP consumption for pH homeostasis (Dechant et al. 2010; Fernández‐Niño et al. 2015; Orij et al. 2009; Stratford et al. 2013; Ullah et al. 2012; Young et al. 2010). In this study, the increased energy demand to maintain a physiological internal pH is indicated by the comparably large consumed carbon fraction from glucose for the production of CO_2_ at external acidic and alkaline pH. Thus, sufficient pH maintenance minimizes the yield of the by‐product CO_2_.

The major difference in metabolite trajectory between constant pH in STR and shifting pH in SF cultivations lies in the pH‐dependent reuptake of organic acids. In STR cultivations, especially acidic pH values reduce organic acid production from the beginning, most likely due to elevated energy demand by pH homeostasis. In SF cultures, the pH drop protonates previously secreted and dissociated organic acids and elevates metabolic burden as mentioned before (Piper et al. 2001). The oxidation of intruding acids could provide additional NADH to support pH homeostasis. However, carbon catabolite repression (CCR) by glucose is expected to suppress the consumption of other substrates, yet *A. oryzae* consumes organic acids despite the presence of sufficient concentrations of glucose. Similarly, *Bacillus subtilis* consumes glucose and malic acid simultaneously, while both substrates induce CCR against succinic acid and glycerol (Kleijn et al. 2010). In contrast, our study reveals the concurrent utilization of glucose and multiple organic acids in *A. oryzae*. Glucose induced CCR inhibits transporters such as the lactate‐pyruvate‐acetate‐propionate transporter Jen1 in *S. cerevisiae* or the maltose ABC transporter in *Lactobacillus casei* (Monedero et al. 2008; Paiva et al. 2009). In our study, the concurrent consumption of glucose and organic acids is attributed to low pH, enabling organic acids to passively diffuse across the cell membrane and hypothetically bypassing transporter‐inhibiting CCR. A similar mechanism has been proposed for *Penicillium ochrochloron*, where the reuptake of organic acids despite high glucose concentrations is attributed to passive diffusion, indicating an equilibrium between extracellular and cytosolic organic acid anions (Artmann et al. 2020). Besides our work, the post‐secretory reuptake of organic acids in *A. oryzae*, especially in production of malic acid, has been observed by other studies too (Brink et al. 2022; Schmitt et al. 2021). Unlike in our results, the reuptake in Schmitt et al. occurs independently of a pH drop. A possible explanation is provided by our cultivations buffered with CaCO_3_, whereby rapidly increasing DOT inhibits the production of malic acid and initiates reuptake. In Schmitt et al. the reduction in volume due to evaporation and sampling potentially lifts oxygen limitation triggering malic acid assimilation (Lotter and Büchs 2004; Maier and Büchs 2001).

To the best of our knowledge, the cyclic release and reuptake of pyruvic acid has not yet been described for *A. oryzae*. However, such a mechanism has been reported for *B. subtilis*, where pyruvic acid has been characterized as an overflow metabolite: malic acid is degraded to pyruvic acid via highly expressed malic enzymes (Jeckel et al. 2023). The excess pyruvic acid is secreted and later reassimilated upon depletion of other carbon sources, accompanied by upregulation of pyruvic acid transporters (Charbonnier et al. 2017; van den Esker et al. 2017). In contrast, our study does not support the overflow metabolite hypothesis for *A. oryzae*, as pyruvic acid is not only secreted during glucose consumption but also reassimilated concurrently. Since pyruvic acid is fully dissociated at neutral pH, passive diffusion across the plasma membrane is unlikely, implying that facilitated diffusion or even active transport may be involved in reuptake. Accordingly, studying the expression of transport proteins could provide mechanistic insights. A direct link to nitrogen limitation appears unlikely, as pyruvic acid secretion in cultures buffered with CaCO_3_ begins after the onset of nitrogen depletion, indicated by the CTR drop, whereas in cultures neutralized with NaOH, pyruvic acid secretion starts before nitrogen depletion. Notably, while pyruvic acid secretion maximizes under neutral to alkaline pH conditions, reuptake occurs only at mildly acidic pH, whereas strongly acidic conditions suppress secretion altogether. This suggests distinct and pH‐dependent mechanisms for release and uptake. At particularly low pH, the decrease in pyruvic acid secretion is possibly caused by increased energy demands for pH homeostasis, with pyruvic acid being oxidized via the TCA cycle to generate reducing equivalents, as suggested by elevated respiratory activity. Further investigation into the mechanisms of pyruvic acid dynamics in *A. oryzae* could include monitoring intracellular pyruvic acid concentrations to determine whether transient accumulation or active metabolic rerouting drives secretion. In addition, the role of intermediates such as oxaloacetic acid as well as the activity of pyruvic acid dehydrogenase and carboxylase, enzymes directing pyruvic acid into oxidative and reductive TCA pathways, respectively, could provide further insights into the metabolic fate of pyruvic acid under varying environmental conditions.

Several hypotheses account for the natural secretion of acids, even surpassing growth limiting levels, including the aggressive acidification of the surrounding medium to suppress growth of competing microorganism (Andersen et al. 2009; Javaid et al. 2019). Others assume organic acid accumulation as an overflow metabolism, storing energy in extracellular consumable metabolites (Foster 1950; Vrabl et al. 2012). Studies from the late to mid‐1900s report anionic acid secretion to provide intracellular charge balance by compensating the lack of H^+^ backflux at alkaline pH (Conway and Brady 1950; Sigler and Höfer 1991). However, in 2020, Artmann et al. suggest a contradicting passively facilitated diffusion of organic acids in *P. ochrochloron* after falsifying the hypothesis of acid secretion for electroneutrality (Artmann et al. 2020). Other theories account the acid production to the solubilization of insoluble nutrients like phosphate and metal containing minerals (Gadd et al. 2014; Mendes et al. 2020). In the context of this study, the reuptake of organic acids suggests the theory of overflow metabolism. However, the reuptake could also belong to other, not energy‐related functions. For example, the degradation of organic acids at acidic pH could act as detoxification of potentially harmful anions. Nevertheless, the formation of citric acid at a particularly acidic pH proves to be contrary to this hypothesis.

Citric acid is produced in STR cultivations only at low pH and in SF cultures it is exclusively formed following a pH drop. A similar phenomenon is reported in *A. niger*, predominantly producing citric acid under acidic pH conditions (Lingappa et al. 2007; Prasad et al. 2013). Various hypotheses have been proposed to explain citric acid accumulation by *A. niger* in acidic milieu. One example includes an increase in binding affinity of mitochondrial citric acid transporters relative to aconitase (Kubicek 1987). Another hypothesis suggests citric acid accumulation results from reduced activity of aconitase and isocitric acid dehydrogenase; this explanation is weakened by reports indicating an intact TCA cycle during citric acid production (Agrawal et al. 1983; Jernejc et al. 1992; Kubicek and Röhr 1985). Previously mentioned hypotheses assume a shift in cytosolic pH due to extracellular acidification, triggering metabolic changes. This assumption conflicts with the well‐documented pH homeostasis typically maintained in *Aspergillus* species (Hesse et al. 2002). In the presence of stressors like nitrogen depletion, the integrity of pH regulation may be compromised, thereby increasing the plausibility of hypotheses that involve cytosolic acidification. While previous hypotheses focus on enzyme inhibition due to cytosolic acidification, transcriptome data and the identification of pH‐regulated secondary metabolite clusters suggest that citric acid production in *A. niger* may instead reflect a transcriptionally mediated response to acidic pH (Andersen et al. 2009). A further contributing factor, compatible with intact intracellular pH homeostasis, involves acidic extracellular pH facilitating ATP turnover via plasma membrane H^+^‐ATPases, thereby potentially preventing metabolic imbalance during citric acid production (Karaffa and Kubicek 2003). While these hypotheses do not directly explain citric acid production, they may describe physiological conditions that enable sustained acidogenesis. Further studies will be essential to elucidate the regulatory mechanisms underlying pH‐dependent citric acid production.

A direct consequence of the pronounced pH dependency of organic acid production is the necessity to maintain precise pH homogeneity during scale‐up. In large cultivation systems, prolonged mixing times risk local pH gradients, causing short‐term metabolic shifts in *A. oryzae* and potentially reducing malic acid yield in favor of citric acid formation (Lara et al. 2006). Demand‐based addition of neutralizing agents offers the advantage of minimizing excess dosing and tailoring pH levels; however, its success depends on rapid and uniform distribution throughout the reactor volume. Complementary to optimized mixing setups, distributed feed systems ensure consistent and spatially resolved delivery of neutralization solutions by targeting zones of high turbulence within the reactor (Namdev et al. 1992). In contrast, excess CaCO_3_ is uniformly pre‐distributed throughout the cultivation medium, obviating the need for complex dosing infrastructure and offering operational simplicity and reliability.

## 5 Conclusion

The results presented here show for the first time the comparable use of alkali or alkaline earth hydroxides and carbonates to produce organic acids using *A. oryzae*. The use of hydroxides instead of carbonate‐containing neutralizing agents allows unambiguous determination of microbial CO_2_ release, enabling both sensitive detection of metabolic shifts and accurate carbon balancing. In addition, the study demonstrates that *A. oryzae* produces considerable amounts of malic acid when neutralized with Na^+^‐, K^+^‐, Ca^2+^‐, or Mg^2+^‐based agents. K^+^ stimulates citric acid formation. Moreover, acidic pH stimulates the production of citric acid, whereas neutral pH promotes the production of organic acids associated with the rTCA branch. Particularly valuable for pH‐dynamic production processes, the data demonstrate the recirculating production and consumption of organic acids as a function of pH. Finally, evidence suggests that excess CaCO_3_ buffering induces hypoxia, thereby stimulating malic acid production. This study serves as a basis for the optimization of future processes as well as for the re‐evaluation of previously published studies.

## Author Contributions

**Lukas Hartmann:** conceptualization, investigation, methodology, formal analysis, visualization, writing – original draft, writing – review and editing. **Mark Christopher Martin:** conceptualization, investigation. **Anke Neumann:** conceptualization, supervision, project administration, writing – review and editing. **Dirk Holtmann:** conceptualization, supervision, resources, writing – review and editing. **Katrin Ochsenreither:** conceptualization, supervision, funding acquisition, supervision, writing – review and editing.

## Conflicts of Interest

The authors declare no conflicts of interest.

## Supporting information


**Supplement 1:** Ammonium and CDW of *Aspergillus oryzae* DSM 1863 cultivations with constant pH in STR (A, B), pH of cultivations buffered with excess CaCO_3_ buffer (C), CDW of cultivations with different neutralizers (D), CDW and oxalic acid concentrations of SF cultivations (E, F). **Supplement 2:** 2D scans of *Aspergillus oryzae* DSM 1863 in STR cultivations with (A) different neutralizer and (B) varying pH in STR cultivations. **Supplement 3:** Yield of carboxylic groups from alkali equivalents of *Aspergillus oryzae* DSM 1863 cultivations with different neutralizers (A) and constant pH (B) in STR. **Supplement 4:** 2D scans of *Aspergillus oryzae* DSM 1863 in SF cultivations with different buffer concentrations. Images were taken after cultivation.

## Data Availability

The data that support the findings of this study are available from the corresponding author upon reasonable request.
