# Supplementary material for: Understanding the Role of pH Regulation and Neutralizing Agents in Organic Acid Production and Growth of Aspergillus oryzae
Source: Biotechnol Bioeng. 2025 Oct 31;123(1):116–33. doi: 10.1002/bit.70091 (PMC12699135; doi:10.1002/bit.70091)
Supplement: Supplementary file 1 — Supplement 1: Ammonium and CDW of Aspergillus oryzae DSM 1863 cultivations with constant pH in STR (A, B), pH of cultivations buffered with excess CaCO3 buffer (C), CDW of cultivations with different neutralizers (D), CDW and oxalic acid concentrations of SF cultivations (E, F). Supplement 2: 2D scans of Aspergillus oryzae DSM 1863 in STR cultivations with (A) different neutralizer and (B) varying pH in STR cultivations. Supplement 3: Yield of carboxylic groups from alkali equivalents of Aspergillus oryzae DSM 1863 cultivations with different neutralizers (A) and constant pH (B) in STR. Supplement 4: 2D scans of Aspergillus oryzae DSM 1863 in SF cultivations with different buffer concentrations. Images were taken after cultivation. [file BIT-123-116-s001.docx]

**Understanding the Role of pH Regulation and Neutralizing Agents in Organic Acid Production and Growth of *Aspergillus oryzae***

Supplements

Supplement 1 Ammonium and CDW of *Aspergillus oryzae* DSM 1863 cultivations with constant pH in STR (A, B), pH of cultivations buffered with excess CaCO_3_ buffer (C), CDW of cultivations with different neutralizers (D), CDW and oxalic acid concentrations of SF cultivations (E, F). Concentrations for ammonium consider volume adjustments by evaporation and neutralizer but do not account for loss by sampling. CDW includes fungal biomass as well as incorporated insoluble particles. Dashed lines and Akima-spline-connected standard deviations are provided for visual guidance.


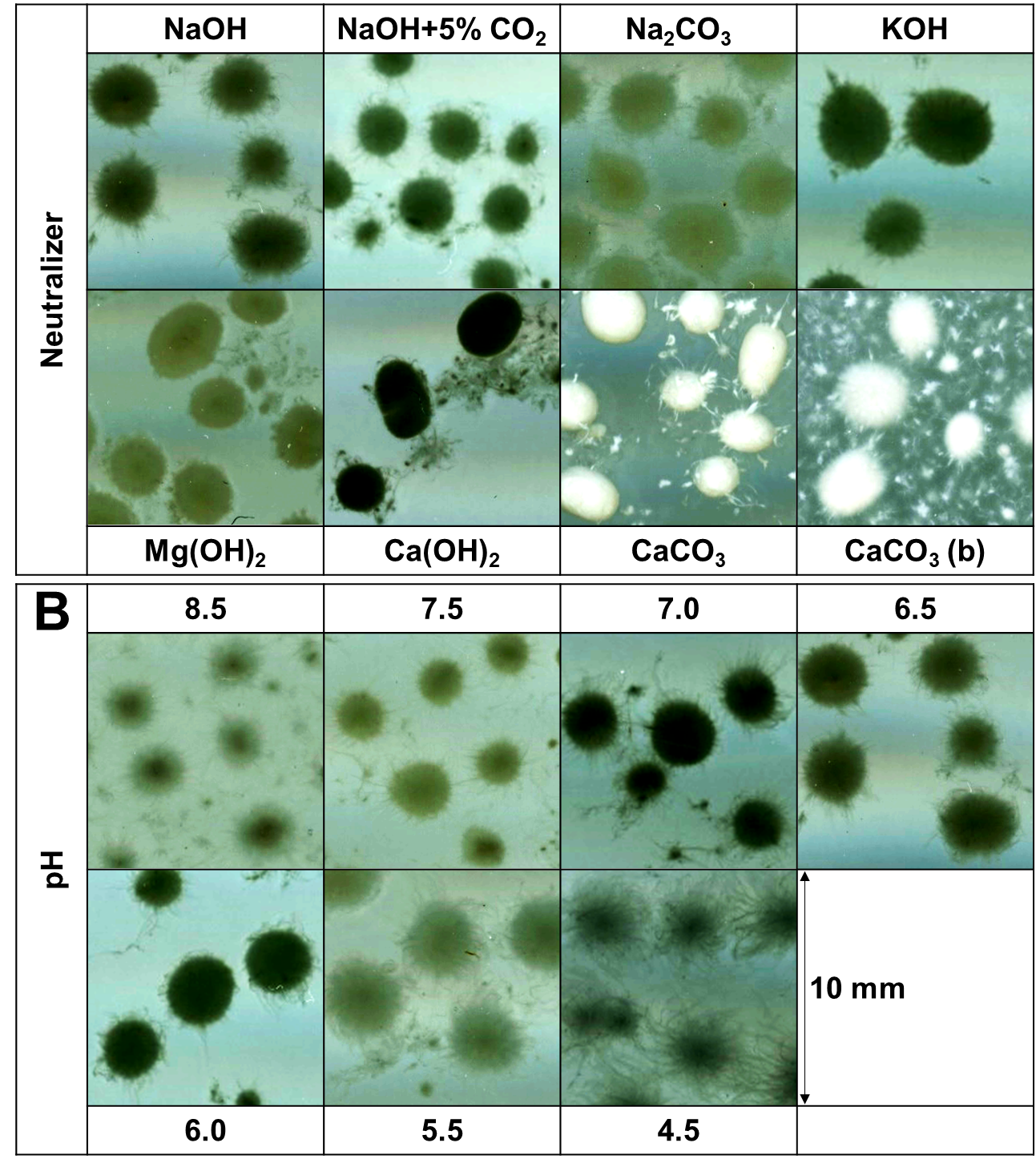


Supplement 2 2D scans of *Aspergillus oryzae* DSM 1863 in STR cultivations with (A) different neutralizer and (B) varying pH in STR cultivations. All images represent biomass harvested after 2 d of cultivation. Contrast and brightness of the images are adjusted to enhance visibility. All images are processed using the same settings.

Supplement 3 Yield of carboxylic groups from alkali equivalents of *Aspergillus oryzae* DSM 1863 cultivations with different neutralizers (A) and constant pH (B) in STR. The yield of produced carboxylic groups was calculated by dividing the total molar amount of quantified carboxylic groups by the molar equivalents of consumed alkaline equivalents, including hydroxides and carbonates. Carbonates were counted as two hydroxide equivalents. The theoretical base demand for neutralizing the consumption of the initial added ammonium was subtracted from the total base consumption. Data indicate that Na^+^-based neutralizing agents result in higher acid yields compared to those based on K^+^, Mg^2+^, or Ca^2+^. For the latter cases, we hypothesize that due to low water solubility of the added alkali, a fraction persists undissolved and therefore does not contribute to neutralization, ultimately lowering the carboxylic acid yield. At acidic cultivation pH, a fraction of the carboxylic groups remains protonated and thus does not require neutralization, thereby increasing the carboxylic acid yield.


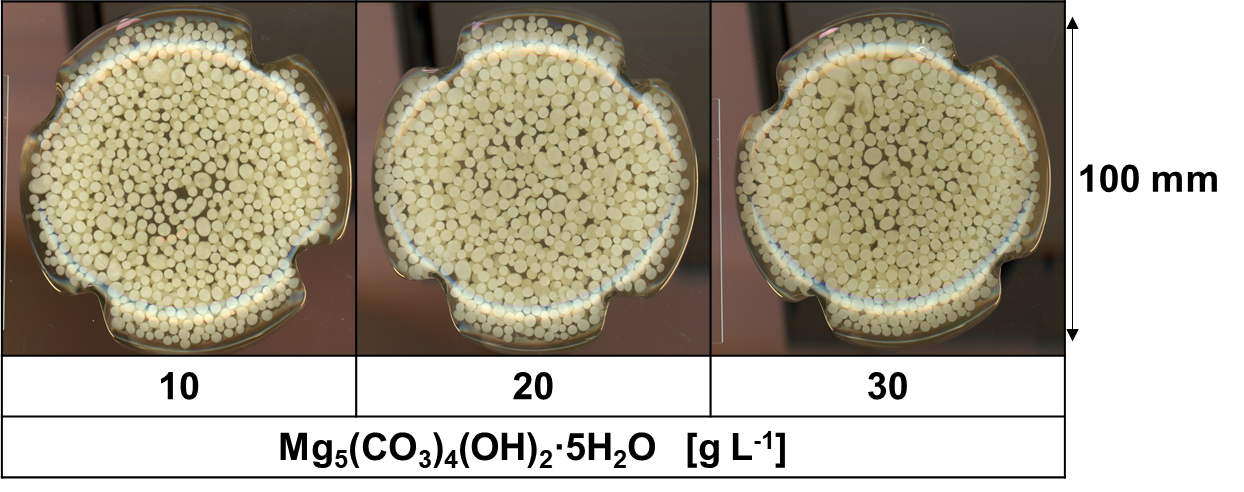


Supplement 4 2D scans of *Aspergillus oryzae* DSM 1863 in SF cultivations with different buffer concentrations. Images were taken after cultivation.
